# Digitally Driven Smile Rehabilitation: A Fully Guided Workflow Using Dual‐Layer Composite Injection Molding

**DOI:** 10.1002/ccr3.71519

**Published:** 2025-11-23

**Authors:** Hiba Hawwa, Ahmed A. Holiel

**Affiliations:** ^1^ Department of Restorative Sciences, Faculty of Dentistry Beirut Arab University Beirut Lebanon; ^2^ Conservative Dentistry Department, Faculty of Dentistry Alexandria University Alexandria Egypt

**Keywords:** composite veneers, digital dentistry, dual‐layer technique, esthetic rehabilitation, guided crown lengthening, injection molding, smile design

## Abstract

A fully digital, guided workflow integrating 3D‐printed reduction guides with dual‐layer composite injection molding enables minimally invasive, biomimetic anterior restorations. By digitally separating dentin and enamel morphology, clinicians achieve lifelike esthetics, translucency, and morphology in a single visit, offering a predictable solution for complex esthetic cases.

## Graphical abstract

Post‐operative views showing (a) natural smile, (b) retracted intraoral view of the maxillary arch, and (c) side view smile.

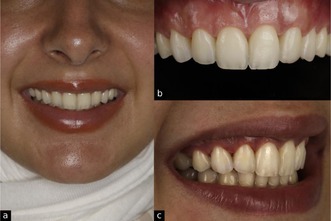

## 1 Introduction

The growing demand for highly esthetic, minimally invasive restorations has driven advances in composite materials and restorative techniques, particularly when integrated into fully digital workflows. Digital protocols enhance precision, efficiency, and predictability while preserving natural tooth structure and minimizing chairside time [1]. Moreover, digital planning facilitates individualized smile design by incorporating anatomical, esthetic, and demographic parameters such as age and gender to optimize treatment outcomes [2, 3].

Direct composite veneers remain a conservative, immediate solution for anterior esthetic rehabilitation, typically built using a layered technique to replicate dentin morphology and enamel translucency [4]. While effective, this method is highly technique sensitive. Indirect restorations offer superior control over form and esthetics but involve higher costs, multiple visits, and technical complexity [5]. Fully digital injection techniques provide a hybrid approach, combining digital accuracy with direct clinical application [6].

The flowable composite injection mold technique enables intraoral transfer of digitally planned morphology via a transparent silicone matrix and low viscosity composite, reducing operator dependency and supporting additive, tooth‐preserving restorations [7]. Single‐layer injections simplify the process but may produce homogeneous restorations that lack the depth of natural dentin‐enamel interactions. The two‐layer injection technique overcomes this limitation by sequentially applying a dentin core layer with mamelons, followed by a translucent enamel layer, creating a natural gradient of color and translucency [4, 8]. By integrating digital planning, 3D printing, and layered injection, this approach combines the precision of indirect methods with the efficiency of direct techniques, achieving highly esthetic, biomimetic outcomes with minimal operator variability [1].

Optimal esthetics also require precise soft and hard tissue management [3]. Digitally designed gingivoplasty and tooth preparation guides allow accurate soft tissue recontouring and conservative enamel reduction, ensuring harmonious contours, ideal seating of injectable veneers, and enhanced integration and long‐term stability of restorations [5].

## 2 Case Presentation

### 2.1 Case History and Examination

**FIGURE 1:**
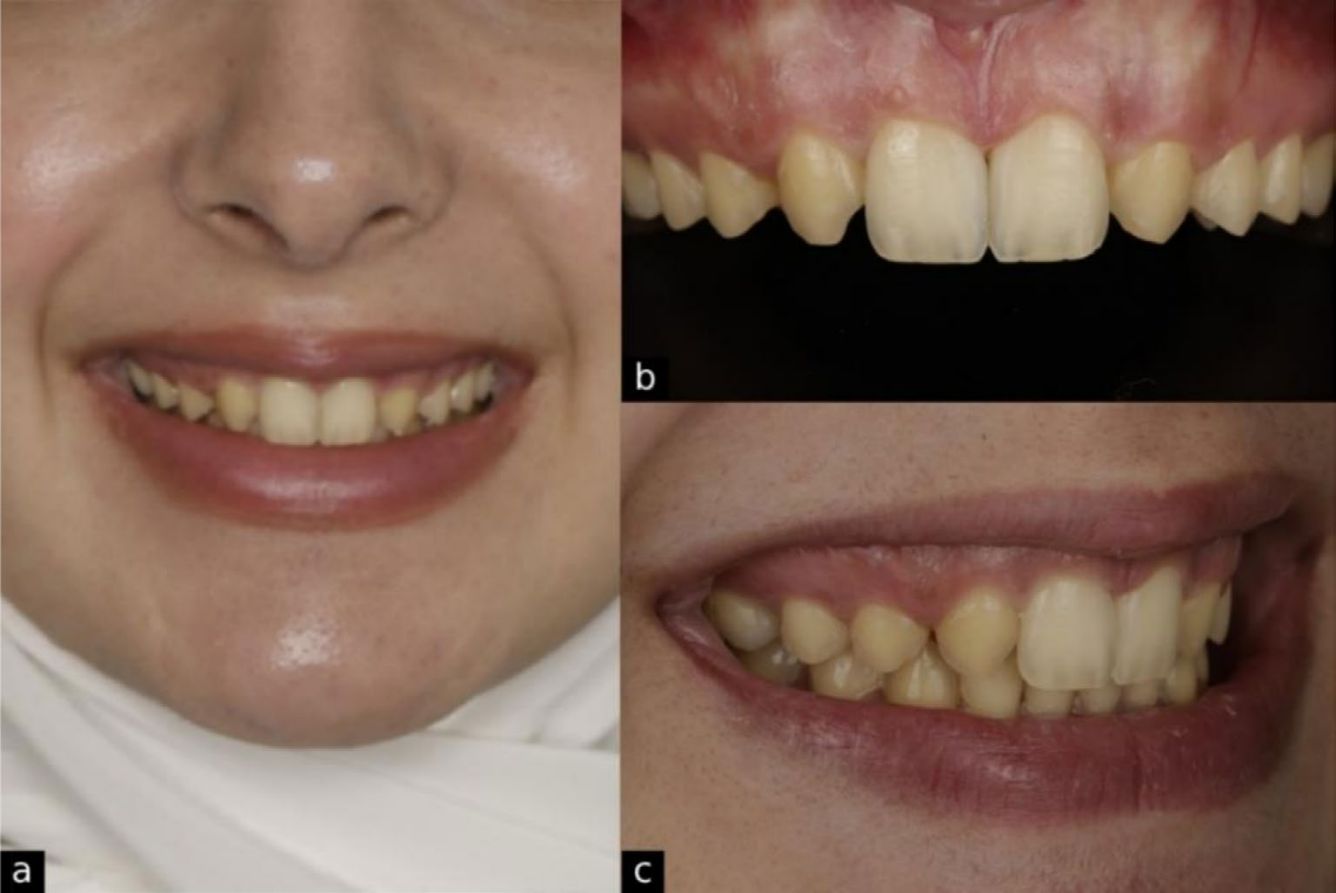
Initial records showing (a) natural smile, (b) retracted intraoral view of the maxillary arch, and (c) side view smile.

A 20‐year‐old female presented dissatisfied with her smile despite recent orthodontic treatment. Her medical history was unremarkable, with no systemic conditions or allergies that would contraindicate dental procedures or affect healing. The patient reported no history of bruxism, temporomandibular disorders, or previous restorative complications. Dental history included routine check‐ups, prophylaxis, and completed orthodontic alignment of anterior teeth. She was concerned about congenitally missing maxillary lateral incisors, excessive gingival display, and small anterior teeth, particularly her canines, which appeared sharp and darker than her central incisors (Figure 1). She wished for the rest of her teeth to match the natural shape, translucency, and esthetic of her central incisors.

Treatment options were discussed with the patient, focusing on restorative solutions to improve the esthetics of her anterior teeth. These included conventional direct composite veneers to reshape the canines and anterior teeth, indirect ceramic veneers for long‐term esthetic stability, and a digitally guided dual‐layer composite injection molding technique to achieve minimally invasive, precise, and predictable replication of dentin and enamel morphology. After reviewing the benefits and limitations of each approach, the patient opted for the digitally guided dual‐layer injection technique, prioritizing minimal invasiveness, immediate results, and accurate reproduction of natural anatomy.

### 2.2 Treatment

The treatment plan aimed to replicate the appearance and character of the central incisors across the anterior segment for a harmonious smile. A fully digital, guided workflow was used, enabling precise design, layering, and intraoral reproduction with a dual‐layer injection molding technique. Intraoral scanning was performed with the Medit i700 (Medit Corp., Seoul, South Korea), and a digital smile design was created using Exocad software (exocad GmbH, Darmstadt, Germany) to plan tooth proportions and gingival contours. A CBCT scan was used to evaluate bone levels and biologic width, guiding the crown lengthening procedure and ensuring optimal crown height and periodontal stability. The decision regarding soft tissue management was based on the digital smile design (DSD) and CBCT assessment. When excessive gingival display or asymmetry compromised esthetics without violating the biologic width, gingivectomy alone was performed to harmonize gingival contours. However, when the planned correction risked encroaching upon the biologic width, a flap procedure with osseous recontouring was indicated to re‐establish an adequate supracrestal tissue dimension and ensure long‐term periodontal health [3]. The extent of alveolar bone reduction was determined from CBCT cross‐sections according to the digital treatment plan, maintaining approximately 2–3 mm of distance between the alveolar crest and the planned gingival margin to achieve a uniform osseous contour [9].

**FIGURE 2:**
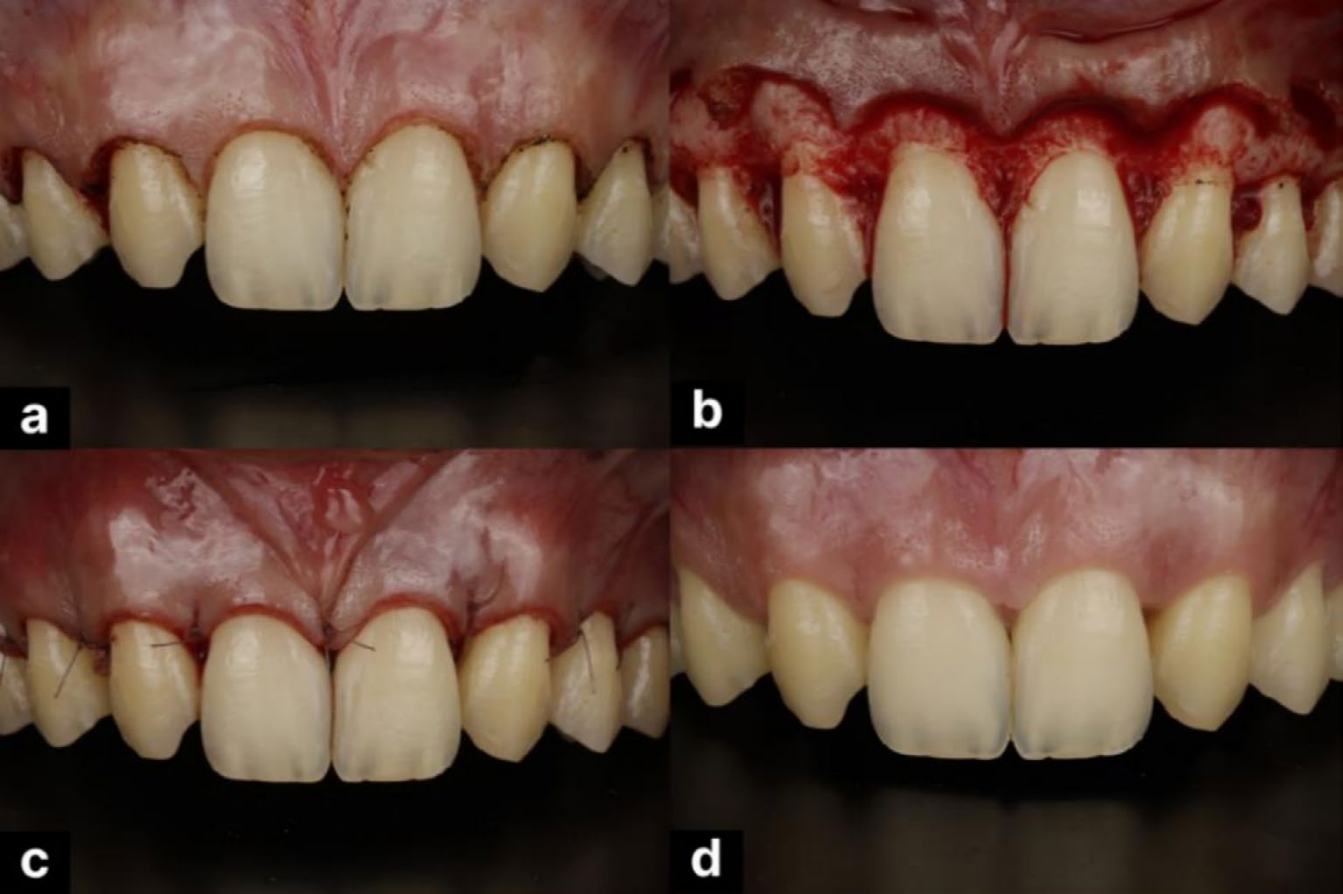
Crown lengthening procedure showing (a) gingivectomy, (b) osteoplasty, (c) suturing, and (d) healing.

**FIGURE 3:**
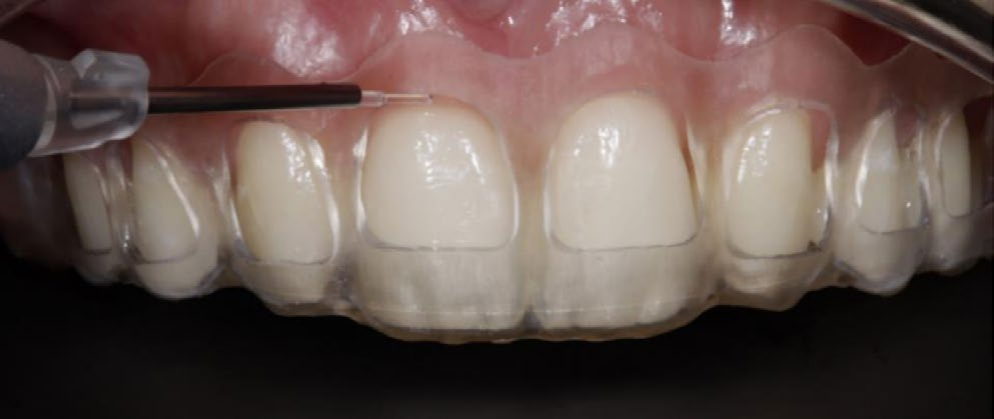
3D‐printed gingivoplasty guide used for precise soft tissue recontouring with a Biolase laser.

The surgical procedure included gingivectomy, alveolar bone recontouring, and flap stabilization using 5–0 Vicryl sutures (Ethicon Inc., Somerville, NJ, USA) (Figure 2). Following adequate healing, a second intraoral scan was performed to capture the updated soft tissue contours. A gingivoplasty guide was then designed in Exocad software, integrating the new scan with the original DSD plan, and subsequently fabricated through 3D printing. This guide was utilized for laser gingival contouring with a Biolase laser (BIOLASE Inc., Foothill Ranch, CA, USA), allowing precise and predictable soft tissue refinement consistent with the planned esthetic outcome (Figure 3).

**FIGURE 4:**
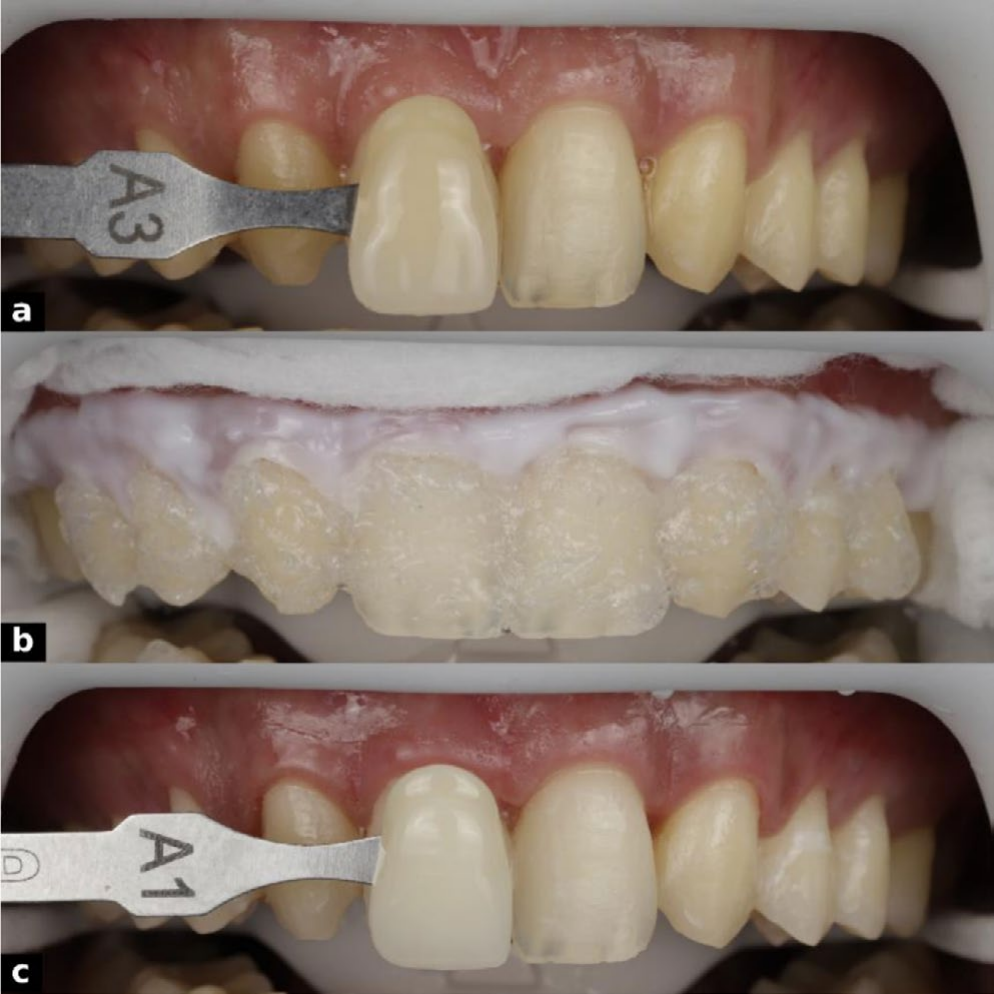
Tooth whitening procedure showing (a) preoperative shade assessment (A3), (b) in‐office bleaching under isolation, and (c) post‐whitening shade verification (A1).

After soft tissue healing, in‐office tooth whitening was performed using the Philips Zoom system (Philips Oral Healthcare, Stamford, CT, USA) in three 15‐min sessions. Retraction was achieved with a mouth retractor, gauze, and cotton rolls, and Liquidam provided gingival isolation and protection. The procedure improved the shade from A3 to A1, creating a brighter, uniform foundation for seamless integration with the central incisors and enhanced smile harmony (Figure 4). Following in‐office bleaching, a waiting period of 2 weeks was observed before composite restorations, allowing color stabilization and recovery of bond strength compromised by residual oxygen radicals [10].

**FIGURE 5:**
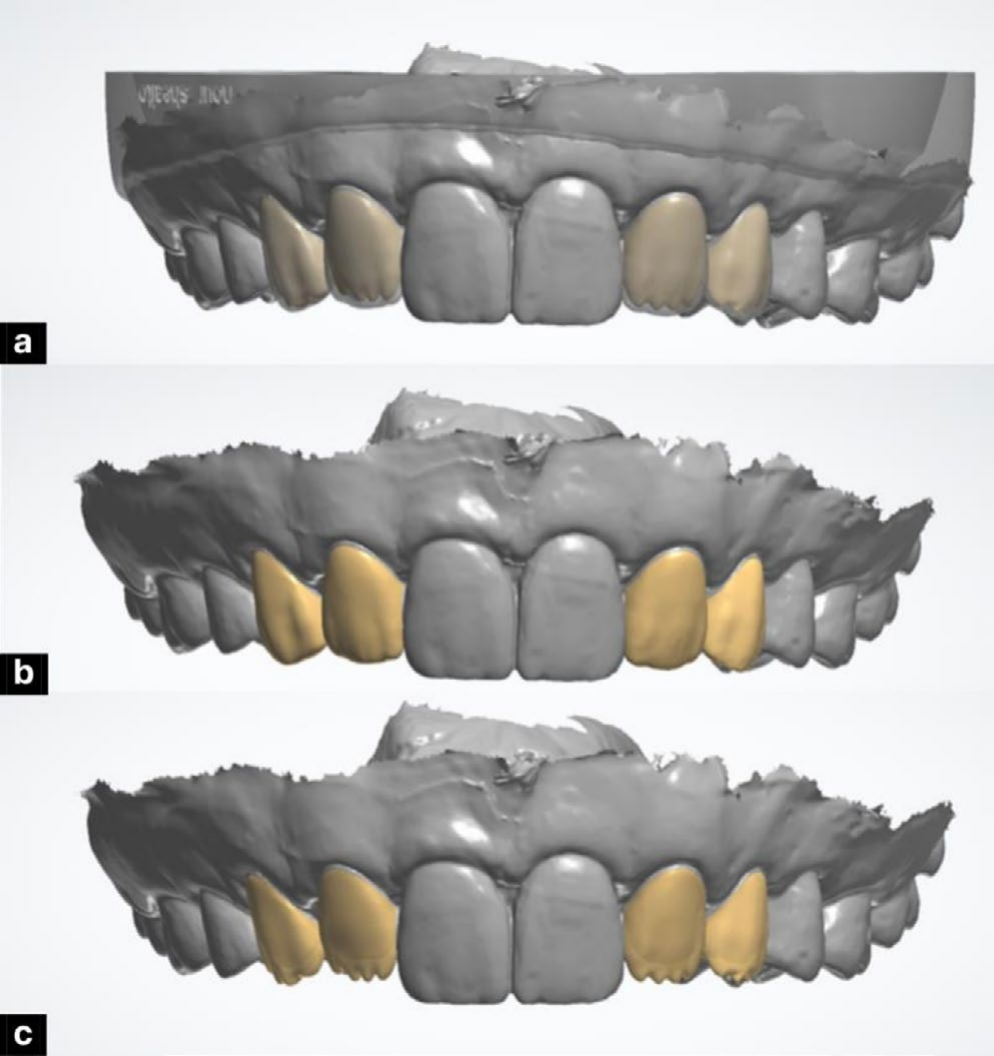
Digital smile design illustrating stratified layering: (a) final enamel design superimposed on dentin, (b) enamel layer design, and (c) dentin layer design.

Composite restorations were planned using a dual‐layer digital design to replicate natural esthetics. The enamel layer was designed first, focusing on external morphology, surface texture, line angles, and incisal edge anatomy, with reduced incisal thickness to enhance translucency. The dentin layer was designed separately, incorporating internal features such as mamelons and primary dentin lobes, guided by the contours of the enamel layer. Both layers were digitally overlaid to assess anatomical relationships and visualize the final outcome (Figure 5). The finalized designs were 3D printed using Dentratec resin (Dentratec GmbH, Frankfurt, Germany), providing a precise and esthetically guided template for dual‐layer composite injection.

**FIGURE 6:**
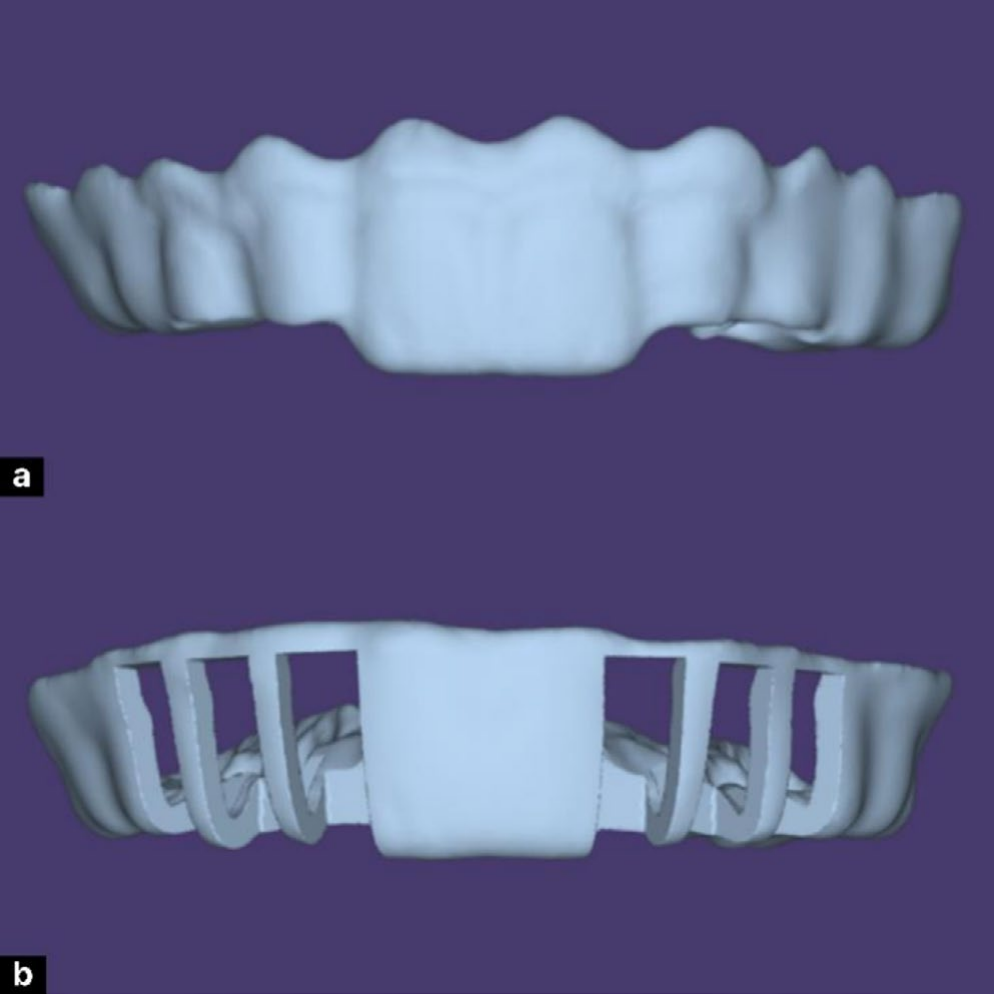
Digitally fabricated reduction guides: (a) incisal reduction guide for precise incisal edge preparation, and (b) buccal reduction guide indicating overall tooth reduction depth.

To enable single‐session preparation and injection, two digitally fabricated reduction guides were created to ensure precise preparation control. A fully digital workflow reflecting the guided minimal‐prep concept was employed. Using Exocad software, a virtual splint was designed, and custom attachments were subtracted to represent the intended preparation depth at specific sites. This process produced: an incisal reduction guide (Figure 6a), aligned with the digital smile design and planned cutback, to direct precise removal of the incisal edges; and a buccal reduction guide (Figure 6b), indicating the required facial reduction depth. Both guides were 3D printed with a Shining 3D printer (Shining 3D Tech., Hangzhou, China) using surgical guide resin, providing stable, accurate templates for conservative and controlled tooth preparation.

**FIGURE 7:**
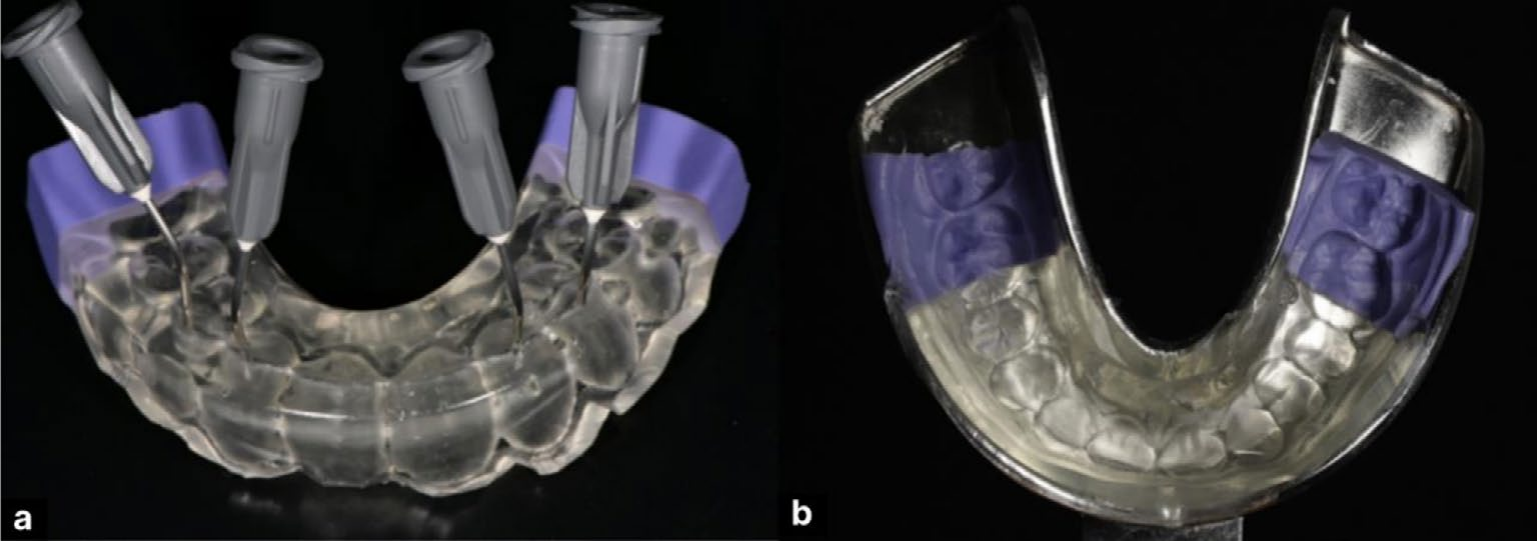
Fabrication of dual‐layer composite molds: (a) enamel mold with injection tips, (b) Exaclear mold in a metal tray after removal of the dentin model.

Transparent silicone indexes were fabricated from the 3D‐printed models by lightly lubricating each model and applying a 2 mm layer of EXACLEAR addition silicone (GC Corporation, Tokyo, Japan). The molds were vacuum processed in a Tecnodent Vacuum Chamber (Tecnodent S.r.l., Bologna, Italy) for 5 min to eliminate bubbles and ensure surface accuracy. After setting, the Exaclear silicone molds were carefully removed from the metal tray and printed models. Each mold was thoroughly inspected to ensure there were no internal air bubbles or defects that could compromise the accuracy or integrity of the design. Proper reduction of the cervical portion of the molds was performed to ensure an optimal and secure fit intraorally, especially when a rubber dam is placed during the procedure. Following inspection and adjustment, injection tips were carefully applied to the enamel mold to create precise channels at the designated injection sites, facilitating controlled material flow during the clinical procedure. The dentin mold was left without any modifications (Figure 7).

**FIGURE 8:**
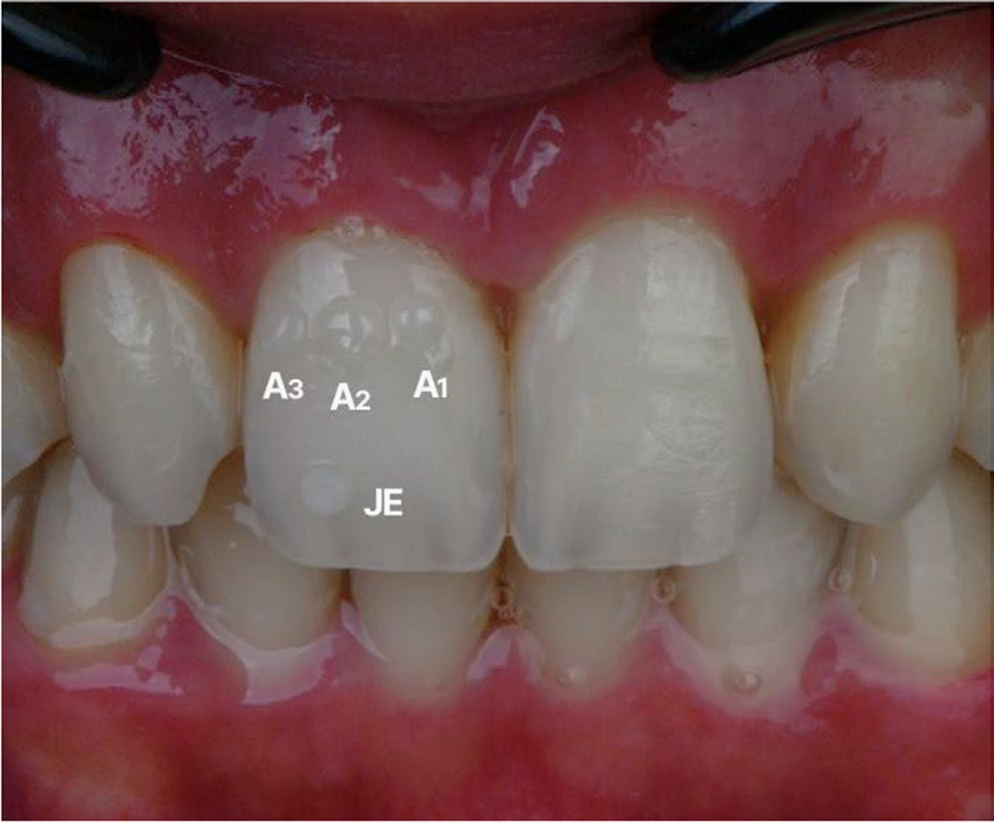
Composite button technique showing dentin and enamel shades for accurate color matching.

**FIGURE 9:**
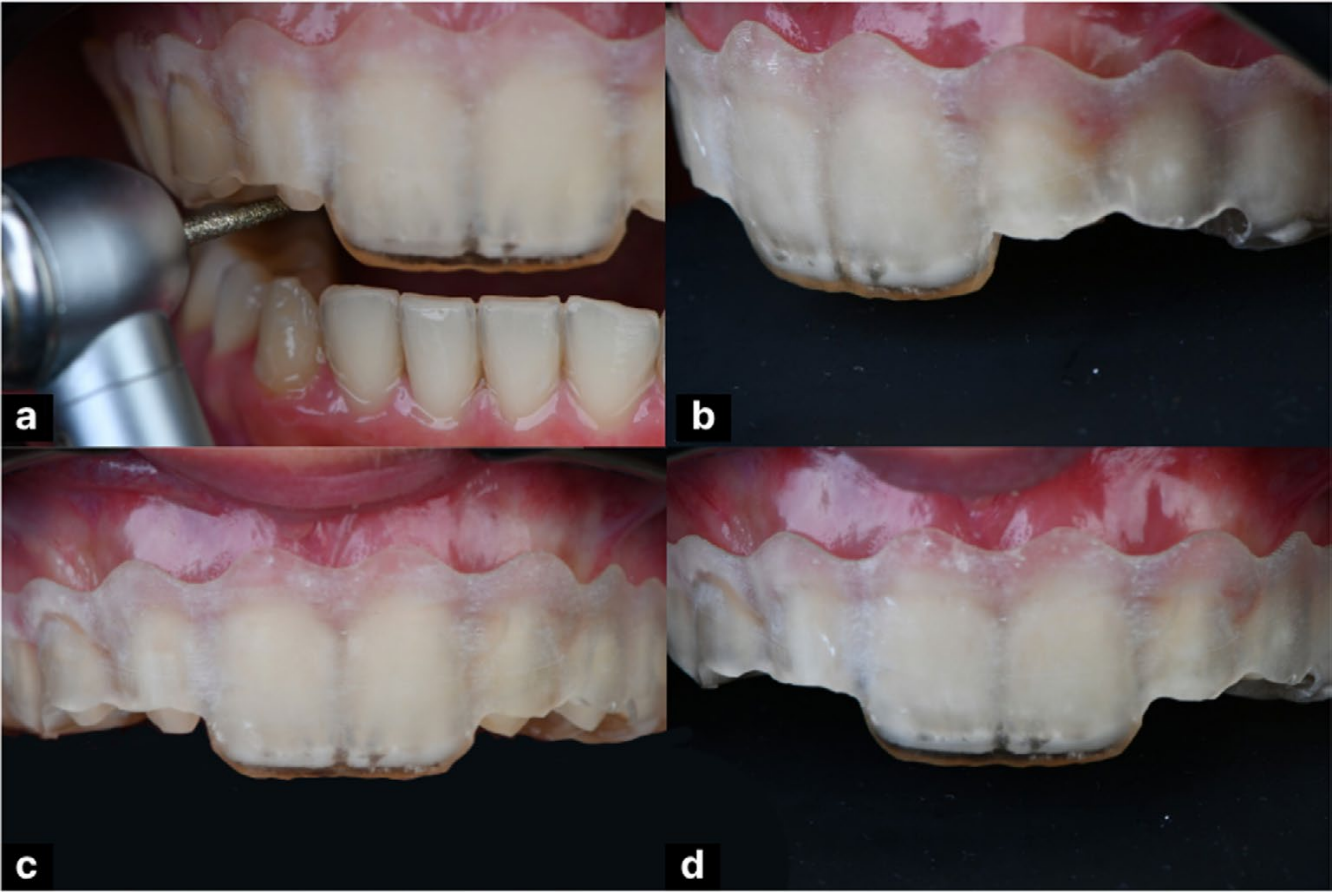
Digital incisal reduction guide and preparation stages: (a) controlled incisal reduction with diamond bur, (b) side view post‐preparation with guide in place, (c) front view before preparation, and (d) front view after incisal reduction.

**FIGURE 10:**
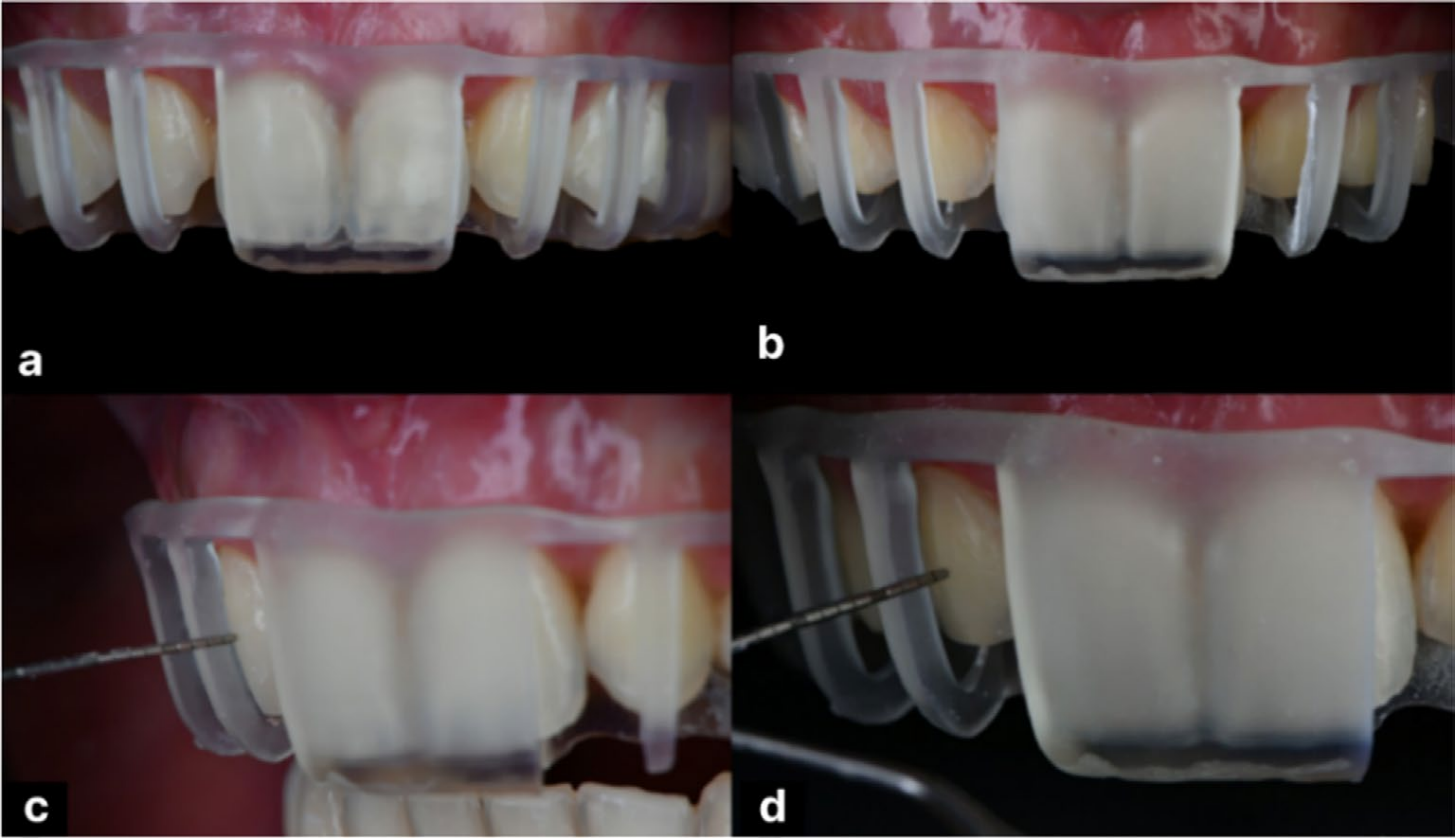
Digital buccal reduction guide and preparation assessment: (a) frontal view before preparation, (b) frontal view after preparation, (c) lateral view with periodontal probe for evaluation, and (d) side view measuring reduction and space for composite injection.

Shade selection was performed by placing and light curing composite buttons on the central incisors to visually match the natural teeth. The composite button technique was performed under controlled lighting conditions and prior to isolation to maintain the teeth in a fully hydrated state, ensuring accurate shade perception. The dentin shade was evaluated by positioning the composite button on the middle third of the tooth, corresponding to the area of greatest chroma and body color, whereas the enamel shade was assessed by placing the button on the incisal third, which best represents enamel translucency and opalescent characteristics [11]. A1 was selected for the dentin layer and Junior Enamel (JE) (GC Corporation, Tokyo, Japan) for the enamel layer to achieve the desired translucency and natural appearance (Figure 8). The incisal and buccal reduction guides were tried intraorally to ensure passive fit. The incisal guide directed precise removal of incisal edges per the digital plan (Figure 9), while the buccal guide confirmed adequate facial reduction (Figure 10). Preparations were performed with fine diamond burs under magnification, maintaining smooth, rounded line angles and preserving enamel to optimize bonding and esthetic outcomes. Guides were used throughout to ensure accurate dimensions for the subsequent dual‐layer injection procedure.

The injection phase began with the isolation of teeth #16 to 26 using a rubber dam, with floss ligatures from #15 to 25 to retract soft tissue and expose preparation margins. Anterior teeth were restored sequentially, protecting adjacent teeth with Teflon tape (Isotape, TDV Dental). Enamel surfaces were selectively etched with 37% phosphoric acid (GC Etchant, GC) for 30 s, rinsed, and air‐dried. A universal adhesive (G‐Premio BOND, GC) was applied actively with a flock‐free micro applicator (ZEROFLOX, Medmix), air‐thinned, and light‐cured for 20 s.

**FIGURE 11:**
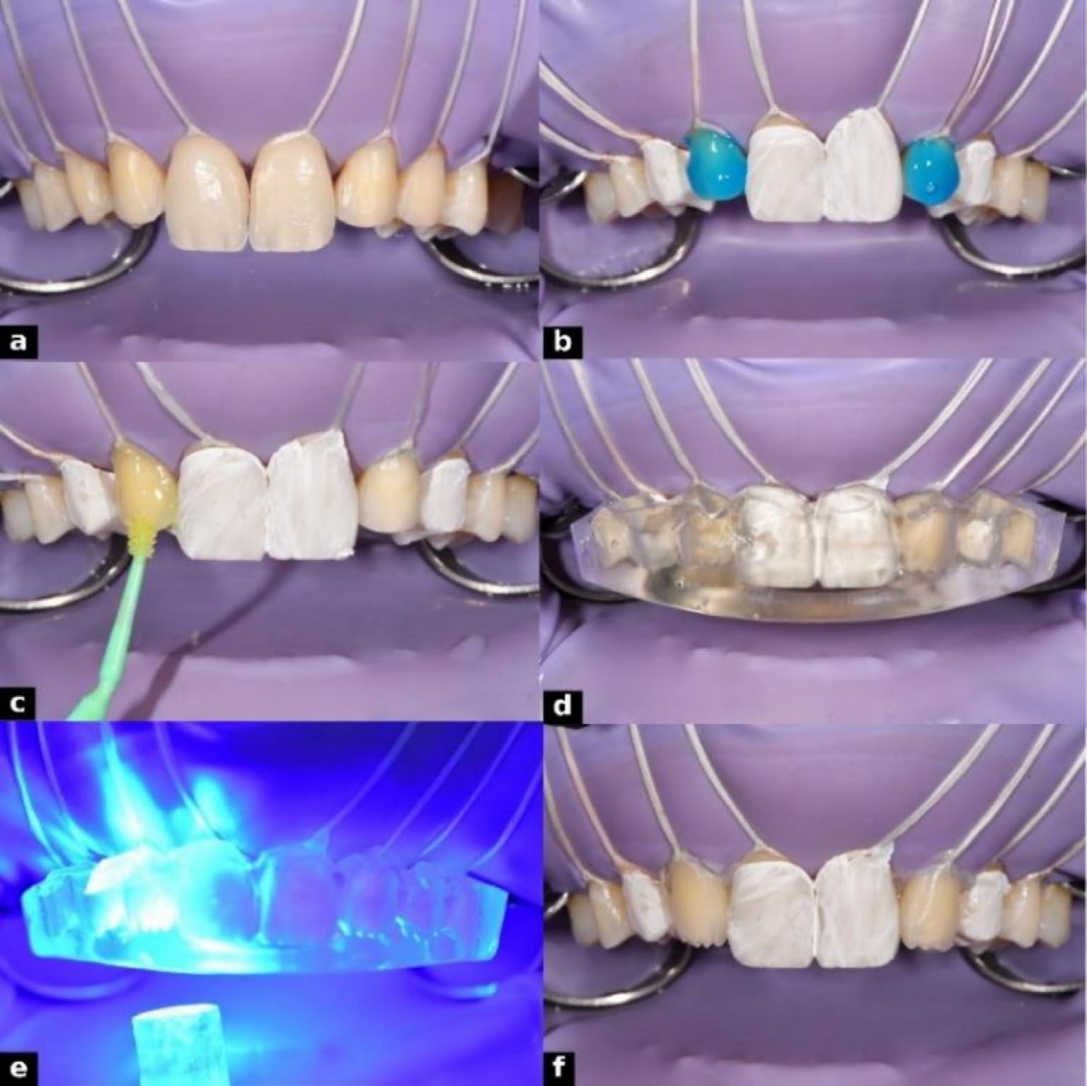
Adhesive protocol and dentin injection steps: (a) isolation with rubber dam and floss, (b) etching with 37% phosphoric acid, (c) application of GC G‐Premio BOND using ZEROFLOX micro applicator, (d) dentin mold placement with composite injection, (e) light curing through the mold, and (f) completed lateral dentin injections.

**FIGURE 12:**
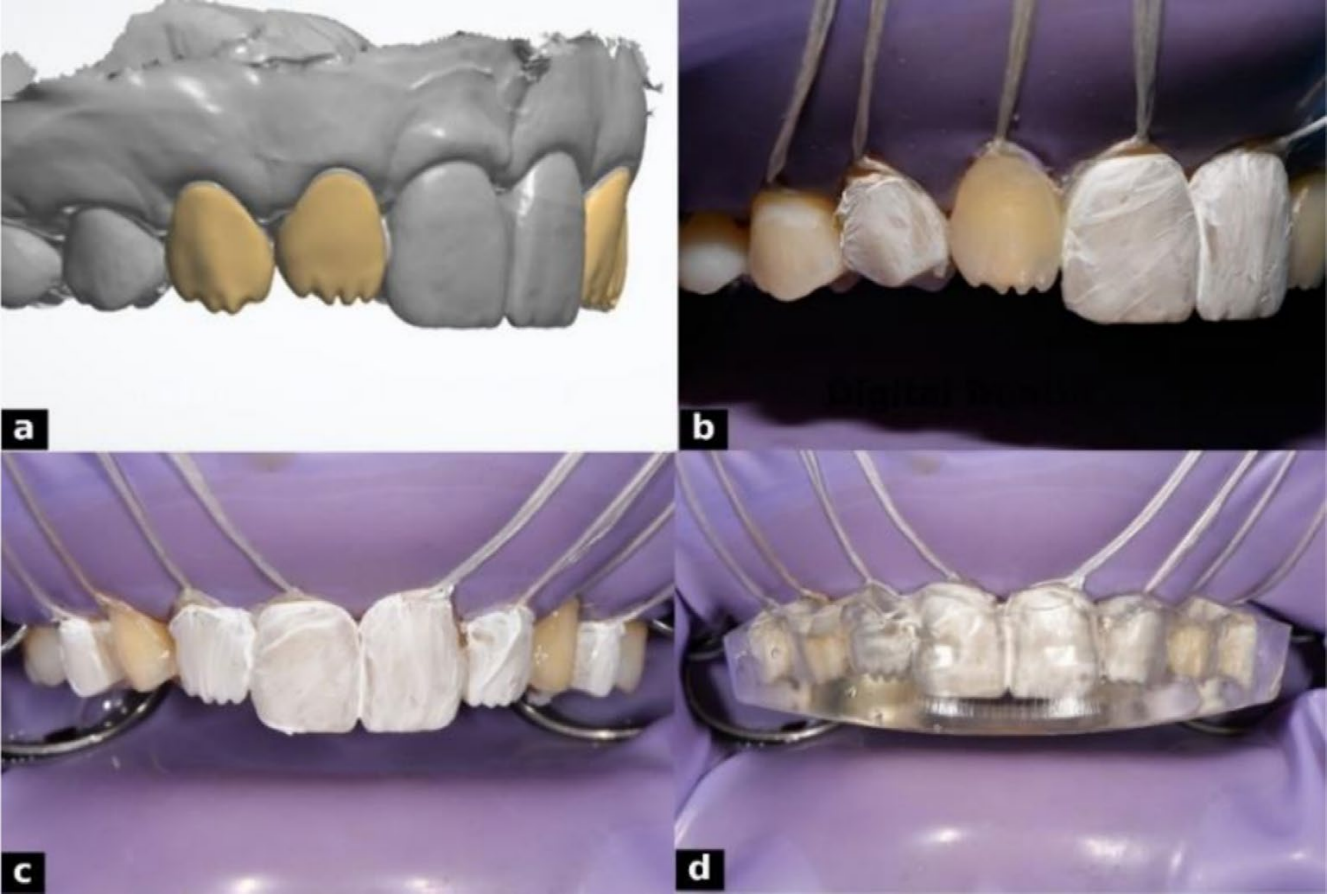
Dentin injection process: (a) digital dentin layer design, (b) lateral dentin injection, (c) Teflon tape isolation of injected surfaces, and (d) mold repositioned for alternating injections.

For the dentin layer, G‐ænial universal injectable flowable composite resin (GC Corporation, Tokyo, Japan; shade A1, Lot No. 2310031) was placed into the silicone index corresponding to the dentin model in an alternating sequence to ensure even distribution. The mold was immediately seated intraorally to prevent air entrapment and ensure complete adaptation. Light curing was performed through the index for 30 s from both buccal and palatal sides. After curing, excess material was trimmed with a scalpel and fine diamond burs (Figures 11, 12). Once dentin injections were completed bilaterally, the procedure proceeded to the enamel layer.

**FIGURE 13:**
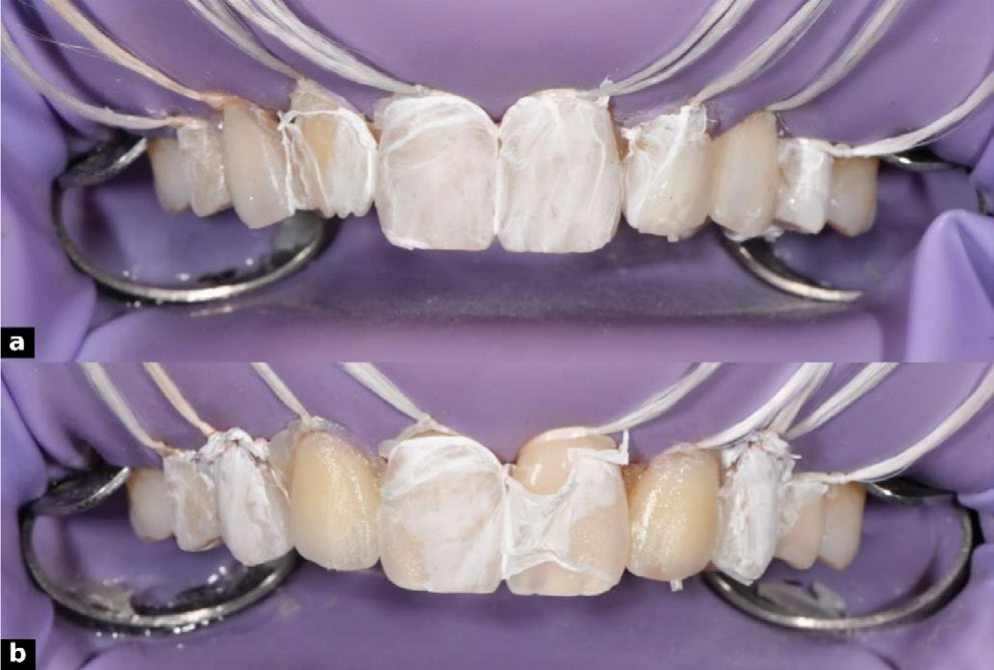
Enamel injection in two alternating steps: (a) first set of enamel injections, (b) second set completing the enamel layer.

For the enamel layer, a more translucent flowable resin, G‐ænial universal injectable (GC Corporation, Tokyo, Japan; shade JE [Junior Enamel], Lot No. 2307051), was used. The enamel silicone index was positioned, and teeth were restored alternately, with adjacent teeth protected by Teflon tape. Care was taken to seat the mold precisely without interference from the dentin layer. Composite was injected to form the outer shell and light‐cured through the index for 30 s from buccal and palatal sides (Figure 13). Excess material was removed from cervical areas and injection sites, ensuring optimal contours and margins. This dual‐layer protocol provided precise control of shade, depth, and surface morphology.

### 2.3 Outcome

**FIGURE 14:**
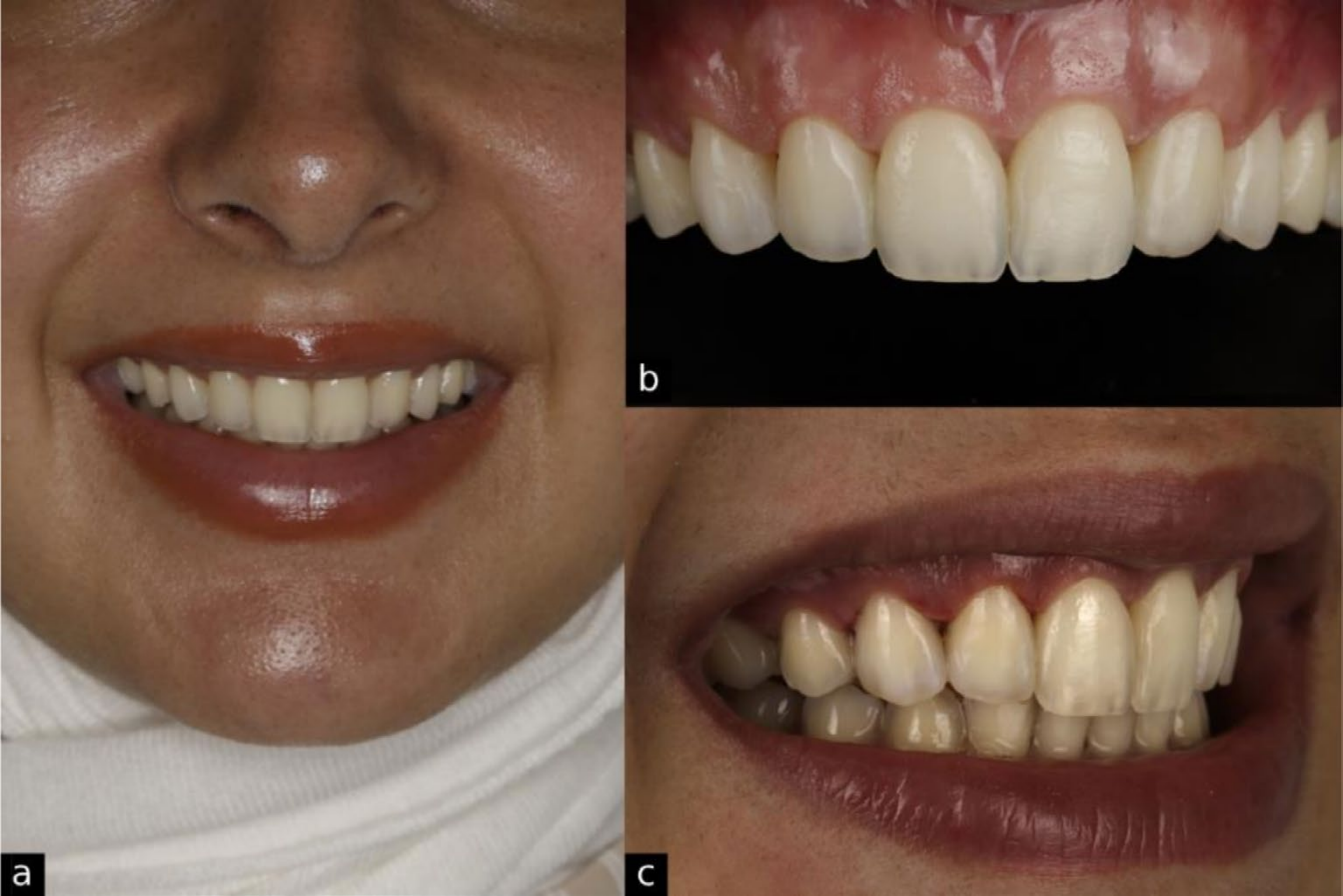
Post‐operative views showing (a) natural smile and facial profile, (b) retracted intraoral view of the maxillary arch, and (c) side view smile.

After completing all restorations, finishing and polishing were performed to achieve a lifelike esthetic result. Abrasive Sof‐Lex XT discs (3M Oral Care, St. Paul, MN, USA) refined contours and transitional lines, while flexible interproximal strips (Epitex, GC) polished contact areas. Finishing and polishing rubbers (EVE Diacomp Plus Twist, EVE Ernst Vetter GmbH) developed natural gloss and texture, followed by high‐luster polishing with a felt wheel and diamond paste (Enamelize, Cosmedent), producing a smooth, enamel‐like surface. The final restorations displayed detailed anatomy and well‐defined enamel texture, accurately replicating the model's features via the EXACLEAR mold, eliminating the need for additional sculpting. Immediate post‐restoration photographs were taken, and the patient expressed high satisfaction with the natural form, shade gradation, and surface texture of her smile (Figure 14).

## 3 Discussion

Direct anterior composite restorations have been revolutionized by digital technologies and advanced flowable materials, enabling precise replication of natural tooth anatomy [12]. In this case, a digitally guided dual‐layer flowable composite injection technique restored esthetics and function in a patient with short crowns, yellow‐toned teeth, and congenitally missing maxillary lateral incisors. The workflow integrated crown lengthening, teeth whitening, and digitally guided tooth reduction, followed by separate enamel and dentin digital designs, 3D printing, and silicone mold fabrication for accurate injection.

Transparent silicone matrices allowed precise transfer of the planned anatomy, reproducing the optical and structural properties of natural teeth. Modern highly filled flowable composites (injectable composites) combine low viscosity with high filler content, high mechanical strength, esthetics, and easy handling, minimizing air entrapment and ensuring optimal adaptation even in complex anatomy [13]. The flowable consistency allows the material to easily adapt and fill the mold, precisely reproducing the planned design. At the same time, they provide high flexural strength, surface hardness, and wear resistance, ensuring long‐term stability [13]. These composites also maintain a smooth surface and high gloss over time, supporting durable and esthetic outcomes in fully guided, minimally invasive workflows [14].

In this case, crown lengthening and digitally guided gingivoplasty ensured ideal gingival architecture [15], while tooth preparation with reduction guides preserved enamel for optimal adhesion. The dual‐layer injection simplified the workflow, minimized technique sensitivity, and achieved lifelike anatomy without extensive sculpting, offering a predictable and efficient alternative to indirect veneers or conventional layering.

The retention of the dual‐layer injectable composite restoration relies on both micromechanical and chemical bonding. At the tooth interface, selective enamel etching and application of a universal adhesive provide strong micromechanical adhesion. At the inter‐layer interface, the oxygen‐inhibited layer of the first (dentin shade) composite allows copolymerization with the subsequent (enamel shade) layer, creating a cohesive, monolithic structure [16]. Digitally guided stratification further reproduces natural dentin and enamel morphology, enhancing macro and micromechanical interlocking. This biomimetic approach, combined with minimally invasive preparation, optimizes retention, durability, and enamel preservation.

Fully guided workflows provide high precision, predictable esthetics, and reduced operator dependency; however, they also present limitations compared with conventional direct layering techniques, including higher cost, longer planning and fabrication time, reliance on digital skills and equipment, and limited potential for intraoral adjustments once guides and molds are fabricated.

While immediate results demonstrated excellent esthetic and functional outcomes, long‐term success requires maintenance to preserve surface gloss, marginal integrity, and color stability. With careful case selection, understanding of material limitations, and regular follow‐up, the dual‐layer flowable injection technique provides a minimally invasive, precise, and highly esthetic solution for anterior restorations.

## 4 Conclusion

A digitally guided workflow combined with a dual‐layer injectable composite technique enabled precise, minimally invasive restoration of short, yellow‐toned teeth with congenitally missing lateral incisors. Separate digital designs for dentin and enamel, along with 3D‐printed models and silicone molds, ensured anatomical accuracy and lifelike esthetics. Immediate results demonstrated excellent form, shade, and surface texture, while long‐term maintenance of color and gloss should be monitored. This approach offers a predictable, efficient, and esthetically superior alternative to conventional multi‐layered techniques, highlighting the potential of fully digital, biomimetic restorative workflows in complex anterior cases.

## Author Contributions

**Hiba Hawwa:** conceptualization, investigation, methodology, writing – original draft. **Ahmed A. Holiel:** conceptualization, investigation, methodology, supervision, visualization, writing – original draft, writing – review and editing.

## Funding

The authors have nothing to report.

## Ethics Statement

The authors have nothing to report.

## Consent

Written informed consent was obtained from the patient to publish this report in accordance with the journal's patient consent policy.

## Conflicts of Interest

The authors declare no conflicts of interest.

## Data Availability

The data that support the findings of this study are available from the corresponding author upon reasonable request.
